# Projecting future fluid intake of Chinese children in a warming world

**DOI:** 10.1038/s43856-025-00929-0

**Published:** 2025-06-03

**Authors:** Sui Zhu, Guanhao He, Na Zhang, Yingying Jin, Zhongguo Huang, Shasha Han, Bingxiao Li, Zhiqiang Lin, Fengrui Jing, Fangfang Zeng, Yanhui Gao, Tao Liu, Xiaofeng Liang, Guansheng Ma, Wenjun Ma

**Affiliations:** 1 Department of Public Health and Preventive Medicine, School of Medicine, Jinan University, Guangzhou, China; 2 Department of Nutrition and Food Hygiene, School of Public Health, Peking University, Beijing, China; 3 Department of Neonatology and Pediatrics, The First Affiliated Hospital of Jinan University, Jinan University, Guangzhou, China; 4 Disease Control and Prevention Institute of Jinan University, 601 West Huangpu Avenue, Tianhe District, Guangzhou, China; 5 Jinan University-BioKangtai Vaccine Institute, Jinan University, Guangzhou, China

**Keywords:** Paediatrics, Epidemiology

## Abstract

**Background:**

Adequate water intake is essential for maintaining health, particularly in children and adolescents. In the context of global warming, the likelihood of experiencing more frequent and intense heatwaves increases, posing a serious threat to regions already grappling with water scarcity. Therefore, we aim to explore the exposure-response relationship between ambient temperature and daily total fluid intake (TFI) among Chinese children and adolescents and to forecast their fluid consumption patterns up to the year 2099 in China, considering different climate change scenarios.

**Methods:**

Utilizing data from a 2011 cross-sectional survey of 3713 students (51.98% female) aged 7 to 18 in Beijing, Shanghai, and Guangzhou, this study employs generalized linear mixed models to analyze the association between temperature and fluid intake. Projections of future fluid consumption are made under the Shared Socioeconomic Pathways (SSP) 126, SSP370, and SSP585 scenarios, reflecting a range of possible climate futures.

**Results:**

Our results show a nearly linear relationship between temperature and fluid consumption. For every 1 °C increase, average daily TFI rises by 24 mL (95% CI: 21–27 mL), and plain water intake (PWI) increases by 12 mL (95% CI: 9–14 mL). The daily TFI ranges from 961 mL at 17 °C to 1298 mL at 31 °C. Future projections under different SSP scenarios indicate a substantial increase in fluid intake by the year 2099.

**Conclusions:**

These findings reveal a positive association between ambient temperature and fluid intake with projected increases in hydration needs under future warming scenarios. They highlight important public health implications in the context of climate change and emphasize the need for updated hydration guidelines to protect child health in a warming world.

## Plain language summary

As global temperatures rise and heatwaves become more frequent due to climate change, understanding how temperature affects fluid consumption is important, especially for Children. We explore how ambient temperature influences daily fluid intake among Chinese children and teenagers aged 7–18, using data from over 3700 students in Beijing, Shanghai, and Guangzhou. The results show that fluid intake increases as the temperature rises, with an additional 24 mL of fluid consumed for every 1 °C increase in temperature. Projections indicate that fluid needs will increase significantly by 2099 under different climate scenarios, highlighting the need for public health measures to address hydration in a warming world.

## Abstract (web summary)

Zhu, He, Zhang et al. conduct a cross-sectional study examining the relationship between temperature and fluid intake in Chinese children. Findings reveal a 24 mL daily intake increase per 1 °C temperature rise and projections under climate change scenarios predict substantially higher hydration needs by 2099.

## Introduction

Water is an essential component of human life and accounts for ~60–70% of body mass, playing a crucial role in maintaining optimal health and function of various physiological processes^1^. Both excessive and insufficient water intake negatively impact human health^2^. Adequate water intake sustains fundamental body functions and fosters cognitive alertness, concentration and overall academic performance^3^, which is particularly critical for school-aged children and adolescents in a phase of rapid physical and cognitive growth characterized by increased metabolic rates. Ensuring adequate water intake is essential to support educational progress, participation in extracurricular activities, and overall well-being^4,5^. Therefore, there is a need to establish water intake guidelines and enhance public awareness about the significance of maintaining sufficient hydration. An investigation on water consumption among National Health and Nutrition Examination Survey participants in the United States from 1999 to 2002 revealed an average daily total water intake (including both foods and beverages) of approximately 1.4 L for children and adolescents (aged 4–18 years)^6^. In China, only three surveys have been carried out specifically focusing on total fluid intake (TFI), which encompasses drinking water and all other types of beverages^7–9^. The Chinese Nutrition Society recommends a daily TFI of approximately 1 L for children aged 7–10 years, 1.3–1.4 L for boys aged 11–17 years, and 1.1 to 1.2 L for girls aged 11-17 years^10^.

However, TFI often falls short of adequate intake (AI) recommendations in some countries. Such recommendations usually consider age, sex, activity, and environmental conditions. Prior research has indicated the association between environmental temperature and water consumption^11–13^. For instance, a cross-sectional community study conducted in the US-Mexico population showed a 28% increase in water consumption when outdoor air temperatures exceeded 90 °F^13^. Another study involving Japanese adults revealed that total water intake was about 10% higher during summer compared to winter^14^. During warmer months, individuals exhibit higher water intake to regulate body temperature and replace lost fluids, while cooler months witness comparatively lower water consumption^14^. However, none of these studies have thoroughly investigated the exposure-response relationships between ambient temperature and TFI, and understanding this association will be helpful in assessing how individuals modify their fluid intake in response to different ambient temperatures conditions.

Global warming, primarily driven by anthropogenic greenhouse gas emissions, has raised much concern about its profound impact on Earth’s ecosystems and life forms^15^. The escalating temperatures not only jeopardize water availability but also entail potential shifts in its distribution and the consequent water requirements of individuals^16^. As temperatures continue to rise, the risk of dehydration and its associated health effects could become more pronounced, particularly in regions already facing water scarcity or higher temperatures. Various shared socioeconomic pathways (SSPs) have been defined to simulate future climate change, each depicting diverse socioeconomic trajectories and political contexts encompassing population growth, global inequality, technological advancements, and environmental shifts^17^. Multiple climate change scenarios have been formulated and outlined. By 2099, there is an estimated global temperature rise of ~1.4–4.4 °C under different scenarios^18,19^. Such alterations could increase occurrences of extended heatwaves, intensifying perspiration and fluid loss among individuals. Therefore, more studies are needed to understand the effects of temperature on daily TFI comprehensively and to develop recommendations and policies on fluid consumption further.

To address the relationship between ambient temperature and fluid intake among Chinese primary and middle school students, we use data from a 2011 cross-sectional survey and projected future fluid consumption patterns under various climate scenarios (SSP126, SSP370, and SSP585) by 2099. We find a near-linear positive association, where higher temperatures significantly increase fluid consumption. Future projections under the SSP585 scenario indicate a substantial rise in fluid intake by 2099, reflecting the potential impact of global warming on hydration needs.

## Methods

### Study design and participants

The data were derived from a cross-sectional survey conducted among primary and middle school students during September and October in 2011^7^. A multistage, stratified, cluster-randomized sampling design was conducted to select subjects. First, Beijing, Shanghai, and Guangzhou cities were selected to represent northern, eastern, and southern regions of China, respectively, ensuring geographic diversity. These cities were also included in the 2010 National Adult Fluid Intake Survey, providing a valuable basis for comparative research on fluid intake between children and adults^8^.

**Fig. 1:**
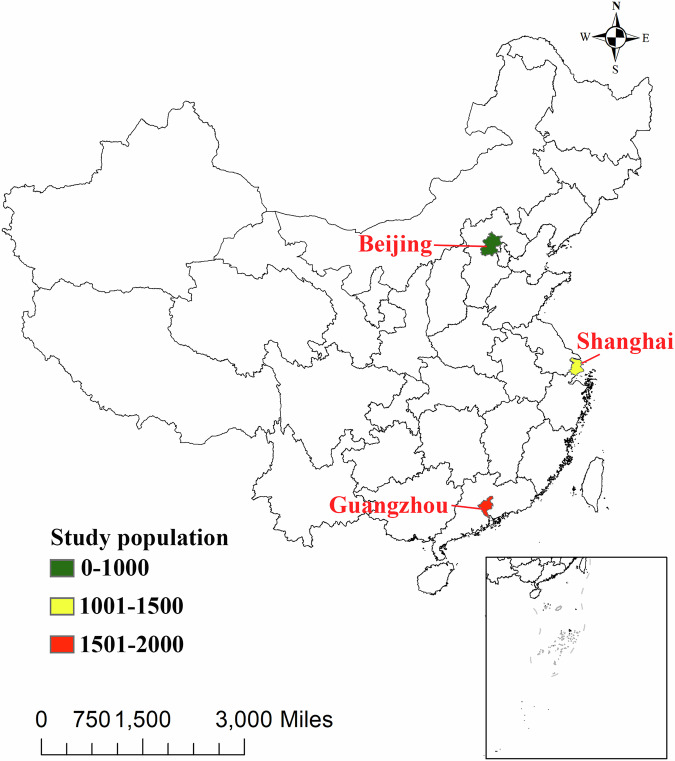
The spatial distribution of the study sample in China. Analyses included *n* = 3713 biologically independent samples. The map shows the locations of Beijing, Shanghai, and Guangzhou, with marker colors representing the size of the study population. The five-pointed star indicates the national capital, Beijing. The scale bar provides distance in miles.

Second, within each city, both an urban and a rural area were chosen by simple random sampling. In the third stage, two primary schools, two middle schools, and two high schools were randomly selected from the designated areas. During the fourth stage, one class from each grade (spanning from grades 3 to 6 for primary schools, grades 7–9 for middle schools, and grades 10–11 for high schools) was selected through a randomized process. Finally, all students aged 7–18 years with good health in the chosen classes (~30 students per class) were included as participants, yielding a total sample size of 4320 individuals across the three cities: 40 students/class × 9 grades × 2 schools × 2 urban-rural areas ×  cities = 4320 participants, as shown in Fig. 1. Students with chronic diseases such as diabetes, hypertension, kidney disorders, or liver disease were excluded from the investigation. Of the final study population, 1783 were males (48.02%) and 1930 were females (51.98%).

The Ethical Review Committee of the Chinese Center for Disease Control and Prevention approved the study protocol (Approval No. 202332). Written consent was obtained from the parents of all participants.

### Meteorological data collection

Throughout the survey duration, the medical practitioners of the designated school recorded indoor or outdoor temperature (°C) and relative humidity (RH, %) at 10:00 AM and 3:00 PM using standardized thermometers and hygrometers^7,20^. A classroom situated on the intermediate levels of the teaching building was chosen to represent climate exposure.

To estimate individual daily exposure to temperature and RH, we first computed the mean values of temperature and RH based on measurements taken every morning and afternoon. Then, time-weighted averaging was used to estimate the individual daily exposure according to the indoor and outdoor activity time of participants and indoor and outdoor temperature or RH (Eq. (1)):

1 $${E}_{j}^{m}={C}_{{in}}^{m}\times \frac{{T}_{{in}}}{{T}_{{in}}+{T}_{{out}}}+{C}_{{out}}^{m}\times \frac{{T}_{{out}}}{{T}_{{in}}+{T}_{{out}}}$$

${E}_{j}^{m}$ refers to the mean individual exposure of subject $j$ at school $m$ to temperature or RH. ${C}_{{in}}^{m}$ and ${C}_{{out}}^{m}$ refer to the indoor and outdoor temperature or RH within school $m$, respectively. ${T}_{{in}}$ and ${T}_{{out}}$ are the age- and sex-specific time spent indoors and outdoors for subjects $j$, respectively, based on data from the 2013–2014 nationwide survey on Environmental Exposure Patterns Among Children aged 6–17 years old in China^21^. This survey, which included 41,439 children across 30 provinces in China, provided representative estimates of typical indoor and outdoor activity times for children^21^.

Projected daily mean temperatures of the three cities during 2015-2099 were obtained from the Coupled Model Intercomparison Project Phase 6 (CMIP6), which was developed by the Inter-Sectoral Impact Model Intercomparison Project (ISI-MIP). We downloaded CMIP6 data from the website of ISI-MIP (https://www.isimip.org/), which was open access. The current study focused on three combination scenarios of SSPs and representative concentration pathways (RCPs), including SSP126, SSP370, and SSP585. SSP126 (SSP1 + RCP2.6) is a sustainable development path at a low level of greenhouse gas emissions; SSP370 (SSP3 + RCP7.0) is a medium-high development path at a mediate-high level of greenhouse gas emissions; and SSP585 (SSP5 + RCP8.5) is a development path ruled by fossil fuels at a high level of greenhouse gas emissions^22,23^.

### Data collection on fluid intake

A 24 h fluid survey was used to collect information on fluid intake over 7 consecutive days, which was a part of a multinational project called Liquid Intake over 7 days (*Liq.In*^7^) conducted with the Chinese Center for Disease Control and Prevention^7,8^. In the week preceding the survey, a standardized water container was distributed to all students, bearing the students’ names and marked with measurement scales. They were instructed to record fluid intake throughout the 7 consecutive days by themselves, including attributes such as type, volume consumed, the location of consumption, as well as the specific commercial names of the consumed liquids during eight distinct occasions, including before breakfast, during breakfast, after breakfast, mid-lunch, after lunch, mid-dinner, after dinner, and during the night. The records were diligently examined daily by the class instructor and the investigator, ensuring meticulous records of the students’ fluid intake.

The volume of TFI was the sum of plain water intake (PWI) and the cumulative intake of all beverages. The sources of PWI encompassed plain boiled water, tap water, mineral water, and purified water. Seven types of fluid intake were classified under the umbrella of beverage intake^7,24^, namely: 1) Milk drinks intake (MDI), including plant-based protein beverages, dairy-based beverages, milk, soy milk, yogurt, and other milk-based drinks; 2) Hot beverages intake (HBI), embracing coffee and tea; 3) Still sugary drinks (SSDs), including conventional tea-based beverages, low-sugar tea-based beverages, sugar-free tea beverages, as well as fruit and vegetable drinks; 4) Carbonated sweet drinks (CSDs), including Cola regular, Cola light/zero and other carbonated soft drinks; 5) Functional beverage intake (FBI), including energy-enhancing and sports-promoting drinks; 6) Cold beverage intake (CBI); and other beverages including traditional Chinses medicine, solidified beverage, and other variants.

The sum of the volumes of CSDs and SSDs culminated in the classification of sugar-sweetened beverages (SSBs) intake, referring to beverages with added artificial sugar or beverages with more than 5% of added sugar content (https://www.hydrationforhealth.com/en/hydration-tools/liqin7/). In this study, we defined the other fluid intake (OFI) to be equivalent to TFI-PWI-SSBs.

### Other covariates

All enrolled students were requested to complete a structured questionnaire covering general information, including identification details (ID), sex, date of birth, grade (primary school, secondary school, and high school), as well as urban or rural residence categorization. Meanwhile, their parents completed the socioeconomic status of students’ families, including monthly household income (<2000 RMB, 2000~, ≥ 4000 RMB). Height in centimeters (cm) and weight in kilograms (kg) were quantified by trained, skilled school health teachers following standardized procedures^9^. Subsequently, the metric of body mass index (BMI) was ascertained through the formula denoting the quotient of weight (kg) over the square of height (m^2^). Based on the value of BMI, the participants were classified into four distinct groups: wasting, standard, overweight, and obesity. The criteria for wasting were determined using an age-specific BMI screening threshold for students aged 6 to 19 years^25^. In contrast, the criteria for overweight and obesity were following guidelines established by the Group of China Obesity Task Force^26^.

Concurrently, participants were directed to complete a physical activity questionnaire to assess the intensity of their physical activities over the preceding week. Moderate-intensity physical activity is characterized as an activity that requires effort but does not feel exhausting, with slightly increased breathing compared to normal. In contrast, high-intensity physical activity refers to activities that require considerable effort, feel challenging, and result in noticeably accelerated breathing compared to normal. The cumulative total physical activity time (minutes/day) is calculated as follows:

Total physical activity time (minutes/day) = moderate-intensity physical activity time (minutes) / number of active days + high-intensity physical activity time (minutes) / number of active days. At last, the physical activity times were stratified into low, moderate, and high levels based on the distribution’s three quantiles.

### Quality control and data validation measures

To ensure the accuracy and reliability of data collection, a comprehensive quality control protocol was rigorously implemented^24^. Investigators were selected based on their professionalism and expertise and subsequently trained by the National Institute for Nutrition and Food Safety, Chinese Center for Disease Control and Prevention. Only those investigators who successfully completed the training and passed competency assessments were allowed to participate in the study. At the beginning of the survey, investigators provided students with thorough and precise instructions regarding the physical activity questionnaire. Students completed the questionnaire under supervision, and forms were immediately collected and reviewed for accuracy, with any issues promptly resolved on-site^24^.

### Statistics and reproducibility

All questionnaires were doubly input into the database by two professional data processors independently, using EpiData 3.1 software. Due to the positively skewed distribution observed in all types of fluid intake consumption (Supplementary Fig. 1), the median and interquartile range (IQR) were used to describe the daily fluid intake per capita. Discrete variables are expressed as frequencies and percentages. Meanwhile, the Spearman correlation coefficients were employed to examine the associations between meteorological factors (temperature and RH) and fluid intake (TFI, PWI, SSBs, and OFI). Because of high correlations between age and grade (${r}_{s}$ = 0.923), we only included the age of students in the primary analysis.

The fluid intake data were repeated measurements, so we used the generalized linear mixed model to explore the associations between temperature and fluid intake in primary and middle school students, which is one of the most common methods in the analysis of longitudinal and clustered data in biological science^27,28^. To explore the nonlinear relationship between temperature and fluid intake, we used a cross-basis matrix representing an exposure-lag-response bi-dimensional function for temperature and the lag time^29,30^, where a smoothing natural cubic spline (*ns*) function of temperature in the cross-basis function with an empirical 3° of freedom (*df*)^31^. Based on exploratory research, the impact of temperature on fluid intake behaviors occurs on the same day, therefore, a simpler model was used. The final model was as follows (Eq. (2)):

2 $$E\left({Y}_{{jt}}\right)=\alpha +{ns}\left({temperature},3\right)+{ns}\left({RH},3\right)+\sum {\beta }_{n}{Cov}+Z\gamma +\epsilon$$

Where ${Y}_{{jt}}$ represents the observed volume of total fluid intake at subject $j$ in day $t$, and *α* is the model intercept. The *ns* function, with an empirical 3 as *df was* used to explore the exposure-response relationship between ambient temperature and fluid intake, while also accounting for potentially nonlinear confounding effects of RH^32^. ${Cov}$ represents other confounders, including age group, sex, BMI, monthly household income, region, intensity of physical activities, cities, and weekday or weekend, and ${\beta }_{n}$ are the regression coefficients. $Z$ represents the random effect with coefficient $\gamma$, and $\epsilon$ represents the error structure.

We also analyzed whether the association between temperature and fluid intake varied by personal characteristics (sex, age group, BMI group, and physical activity), sociodemographic characteristics (region, city, monthly household income), and the day of the week of fluid intake. Based on the exposure-response relationship between temperature and fluid consumption, we also calculated the amount of fluid consumption among students by sex and age group at different temperature levels.

Based on the association between ambient temperature and fluid intake, we could obtain the estimated daily fluid consumption (TFI, PWI, SSBs, OFI) corresponding to each daily mean temperature with 0.1 °C intervals. Combined with the projection daily mean temperature under different SSPs in the future, the daily fluid consumption from September to November in 2015–2099 (the same months as the cross-sectional survey) could be projected. Thus, the average daily fluid consumption across sexes from September to November of 2015–2099 under different SSP scenarios could be projected.

### Sensitivity analysis

A sensitivity analysis was conducted to ensure the robustness of our primary results. First, the *df* for the *ns* function controlling for RH was varied to 2, 4, and 5. Second, we replaced the *ns* function with a linear function in the exposure-response relationship between temperature and fluid intake, maintaining the existing lag structure. Third, outliers were defined as values exceeding 1.5 times the IQR above the upper quartile or below the lower quartile. After the exclusion of these outliers, the sensitivity analysis was repeated to assess the impact of outlier removal on the results.

All results were two-sided, and a *p*-value < 0.05 was defined as statistically significant. We used R software version 4.2.3 with the lmerTest^33^ package to build a distributed generalized linear mixed effects regression model.

### Reporting summary

Further information on research design is available in the Nature Portfolio Reporting Summary linked to this article.

## Results

### Study population characteristics

A total of 3713 students (1783 males and 1930 females) were included in this analysis, with a mean age of 12.18 years. The average time-weighted temperature was 25.4 °C, spanning from 16.11 to 31.07 °C. The proportions of different age groups (7–12 years, 13–15 years, and 16–18 years) were 53.95%, 32.75%, and 13.30%, respectively, but males were similar to females. The cities of Shanghai and Guangzhou represented over 80% of the sample, while Beijing accounted for less than 20% in all age groups. Approximately 70% of students were classified as having normal weight, and the proportion of students in urban regions was slightly higher (59.71%). Notable, the prevalence of intensive physical activities was the lowest, with participation rates below 30%. Other baseline anthropometric characteristics of students were summarized in Table 1.

**Table 1:** The Medians (mL) of daily consumption for TFI, PWI, SSBs, and OFI by the characteristics of students in China

Variables	*N* (%)	TFI		PWI		SSBs		OFI	
		Median	IQR	Median	IQR	Median	IQR	Median	IQR
Total	3713 (100)	1064	679	665	589	71	193	213	250
Sex									
Male	1783 (48.02)	1143	722	750	650	83	224	186	254
Female	1930 (51.98)	999	616	614	528	65	171	227	237
Age group (Years)									
7–12	2003 (53.95)	1003	625	614	505	69	171	214	251
13–15	1216 (32.75)	1137	760	746	665	86	219	207	249
16–18	494 (13.30)	1194	706	800	649	68	204	207	229
BMI group									
Wasting	378 (10.18)	973	642	607	488	71	179	196	220
Standard	2590 (69.75)	1057	658	657	575	71	193	214	247
Overweight	451 (12.15)	1124	678	736	651	71	195	218	266
Obesity	294 (7.92)	1244	832	786	788	73	214	200	280
Region									
Urban	2217 (59.71)	1133	704	717	647	71	201	226	257
Rural	1496 (40.29)	974	624	596	507	71	179	186	225
Physical activity									
Low	1478 (39.81)	1027	634	654	571	71	183	196	242
Moderate	1207 (32.51)	1019	655	637	563	71	186	214	238
High	1028 (27.69)	1191	767	734	650	83	214	233	273
Monthly household income (RMB)									
<2000	194 (5.24)	1084	693	693	592	50	190	157	229
2000–3999	568 (15.34)	1035	704	654	573	71	200	186	229
≥4000	2940 (79.42)	1070	669	670	589	71	193	219	252
City									
Beijing	730 (19.66)	1130	651	712	617	76	214	214	264
Shanghai	1456 (39.21)	1009	628	579	542	79	200	243	248
Guangzhou	1527 (41.13)	1094	719	743	621	64	171	171	231
Weekday									
No	3713 (100)	290	214	171	173	0	71	57	100
Yes	3713 (100)	771	495	500	436	36	129	150	182

*IQR* interquartile range, *TFI* total fluid intake, *PWI* plain water intake, *SSBs* sugar sweetened beverages, *CSDs* carbonated soft drinks, *SSDs* still sugary drinks, *FBI* functional beverage intake, *HBI* hot beverages intake, *CBIs* cold beverage intake, *MDI* milk drinks intake.

### Daily consumption of fluid intake

The median daily TFI consumption was 1064 (IQR: 679) mL, ranging from 214 to 4660 mL (Table 1). A large variation was observed for different types of fluid intake, and the median amounts of PWI, SSBs, and OFI were 665, 71, and 213 mL, respectively. On average, 60% of the TFI consumed was plain water, and less than 8% of the TFI consumed was SSBs. Among male students, those in high school (13–18 years), as well as those involved in more intensive physical activities, demonstrated higher levels of all types of fluid consumption. Students with lower BMI values and residing in rural areas exhibited diminished fluid consumption. Conversely, students from Beijing reported relatively higher fluid water intakes. Other types of fluid intake in different students were shown in Supplementary Table 1.

### Association of ambient temperature with fluid intake

The Spearman rank correlations between meteorological factors and different types of 24-h fluid intake was shown in Supplementary Fig. 2. The correlations indicated that temperature was positively associated with fluid intake, especially with TFI (${r}_{s}$ = 0.12). Meanwhile, PWI was strongly associated with TFI (${r}_{s}$ = 0.75) but negatively associated with SSBs or OFI.

**Fig. 2:**
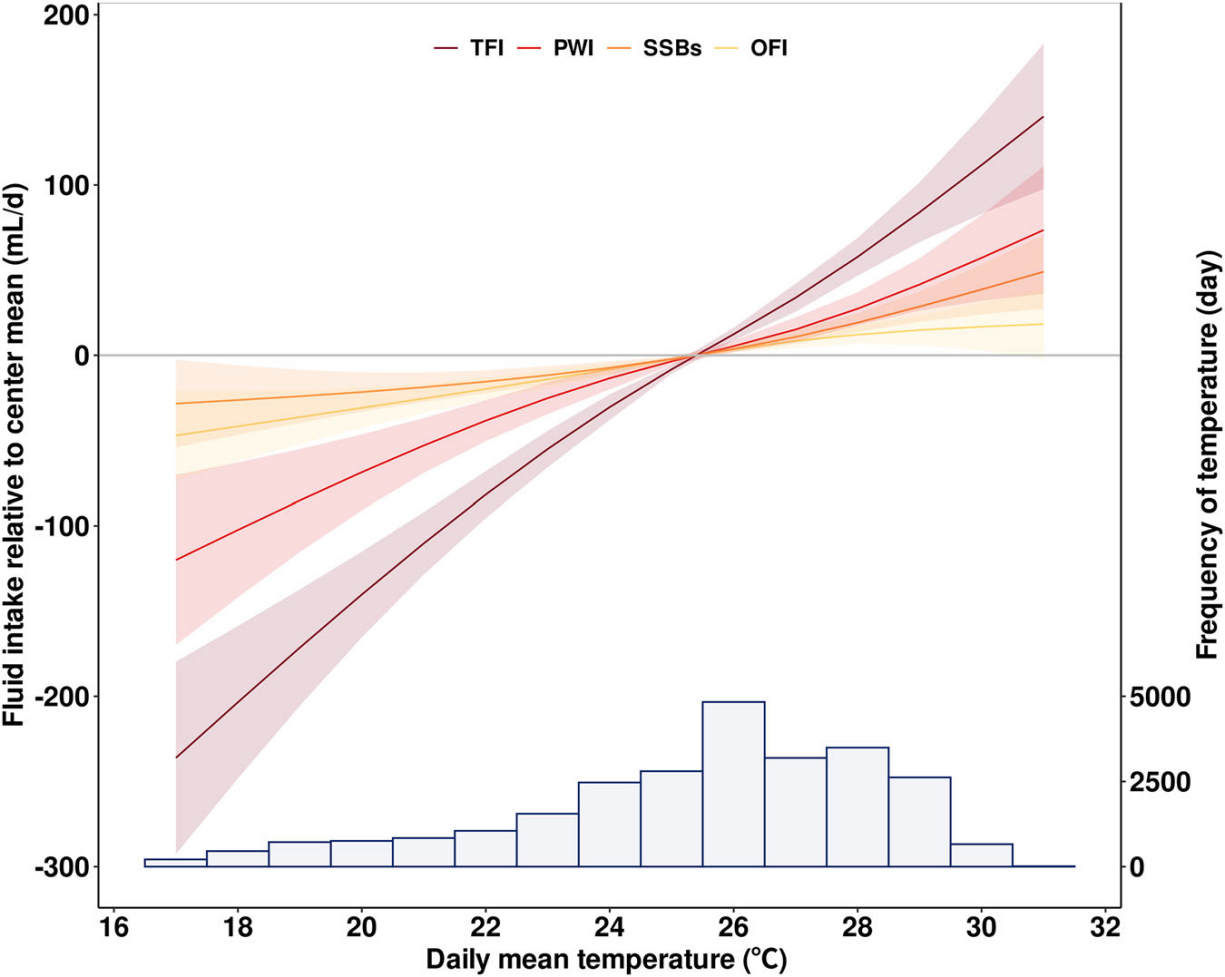
The associations between daily mean temperature and 24 h fluid consumption relative to center mean among children across three Chinese cities. TFI total fluid intake, PWI plain water intake, SSBs sugar sweetened beverages, OFI other fluid intake. All underlying data can be found in Supplementary Data 1; Shaded area denotes the 95% confidence interval of the estimated parameter; The bar graph at the bottom illustrates the frequency distribution of daily mean temperatures recorded during the study period. Analyses included *n* = 3713 biologically independent samples.

Figure 2 shows the relationship between daily mean temperature and different types of fluid consumption. There was a near linearity between temperature and all types of fluid consumption. The slopes were much steeper for TFI and PWI consumption, which indicated stronger associations between temperature and TFI as well as PWI. In contrast, the associations were comparatively weaker for SSBs and OFI. The Spearman rank correlations between meteorological factors and different types of 24 h fluid intake was shown in Supplementary Fig. 2. The correlations indicated that temperature was positively associated with fluid intake, especially with TFI (${r}_{s}$ = 0.12). Meanwhile, PWI was strongly associated with TFI (${r}_{s}$ = 0.75) but negatively associated with SSBs or OFI.

**Fig. 3:**
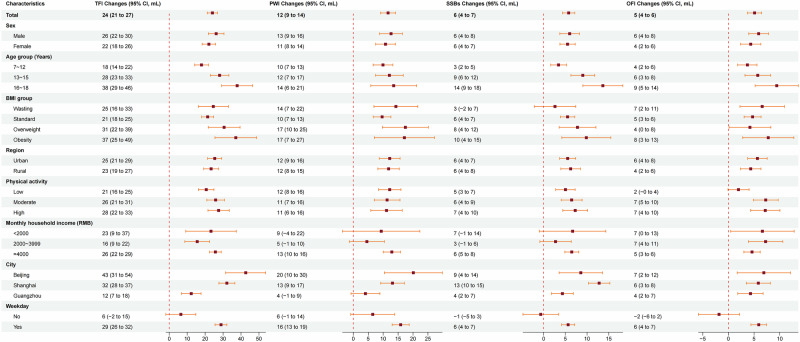
The change of diverse types of fluid intake for each 1 °C rise in temperature among Chinese children by sex, age, body mass index, geographical region, level of physical activity, region, and day of the week. TFI total fluid intake, PWI plain water intake, SSBs sugar sweetened beverages, OFI other fluid intake. Error bars represent the 95% confidence intervals for the estimated changes. Statistical analysis was performed using a generalized linear mixed model; All underlying data can be found in Supplementary Data 2; Analyses included *n* = 3713 biologically independent samples.

Figure 3. presents the effects of temperature on all types of fluid consumption. For each 1 °C temperature rise, daily TFI increased 24 mL (95% CI: 21–27 mL), while daily PWI increased 12 mL (95% CI: 9–14 mL). Specifically, for a 1 °C temperature increase, the highest growth in daily TFI was observed in Beijing, with an increment of 43 mL (95% CI: 31–54 mL), followed by a 12 mL increase (95% CI: 7–18 mL) in Guangzhou. A 1 °C increase on weekdays corresponded to a daily increase of 29 mL (95% CI: 26–32 mL) in TFI. However, compared with weekdays, temperature fluctuations had a much smaller effect on TFI during weekends, as each 1 °C temperature increase resulted in a marginal change of 6 mL (95% CI: −2–15 mL) in fluid intake. A 1 °C increase in temperature increased TFI consumption by 38 mL (95% CI: 29–46 mL) for high school students (16 ~ 18 years), which was ~2.1 times that of primary school students (7–12 years).

### The fluid intake at different temperatures

**Fig. 4:**
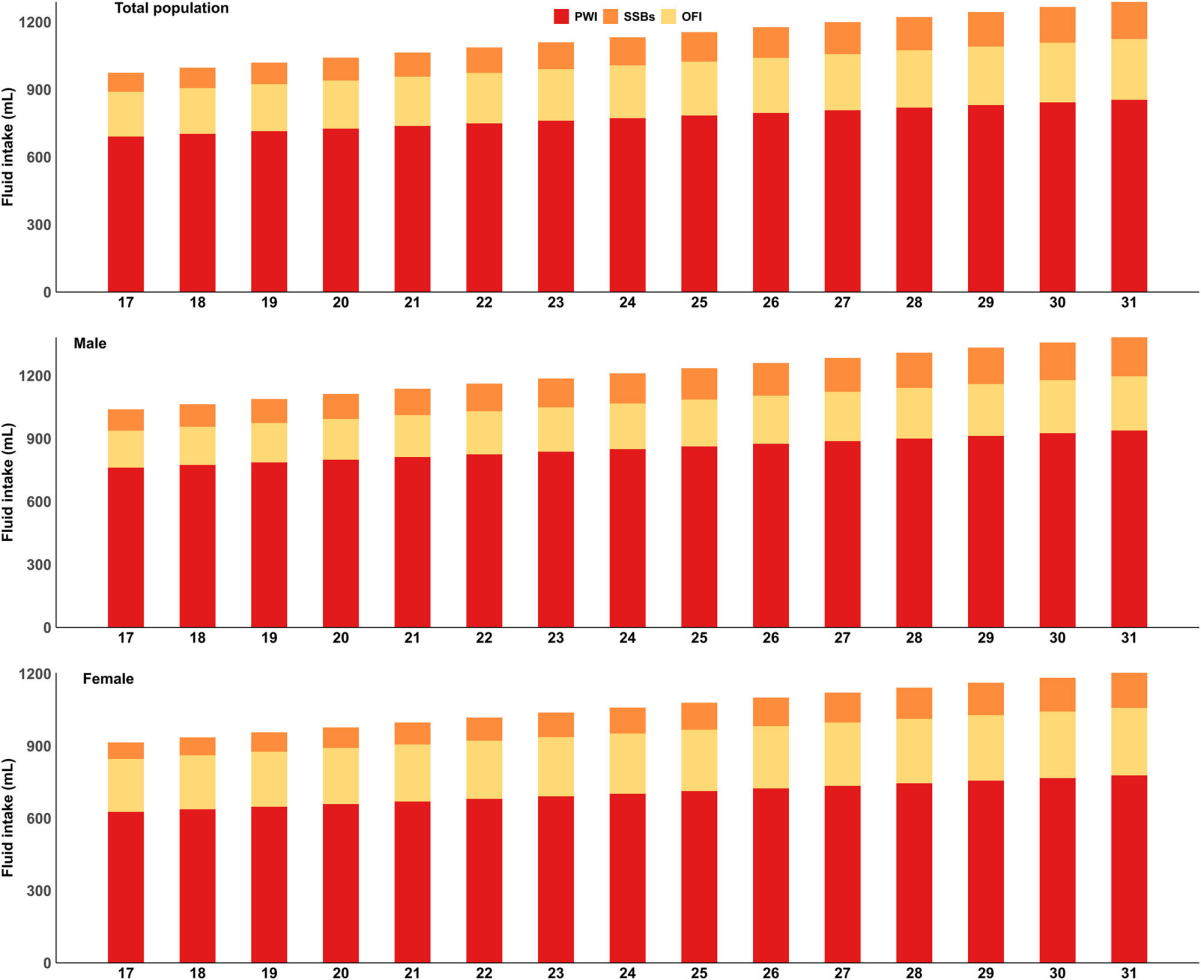
Different types of fluid intake at different temperatures, by sex. PWI plain water intake, SSBs sugar sweetened beverages, OFI other fluid intake. Stacked bar charts illustrate the contributions of different fluid types to total daily intake across temperature ranges; All underlying data can be found in Supplementary Data 3; Analyses included *n* = 3713 biologically independent samples (*n* = 1783 males; *n* = 1930 females).

Figure 4 shows various types of fluid intake at different temperatures (17–31 °C). As the ambient temperature rises, there is a corresponding increase in the consumption of TIF. The minimum TIF consumption was estimated to be 961 mL at 17°C, whereas the maximum TIF consumption reached 1298 mL at 31 °C. PWI was the greatest single contributor to TIF. The lower limit of PWI consumption was ~692 mL at 17 °C, while the upper limit of PWI consumption reached 855 mL at 31 °C. We also observed similar patterns across sexes (Fig.4) and various age groups (Supplementary Fig. 3).

**Fig. 5:**
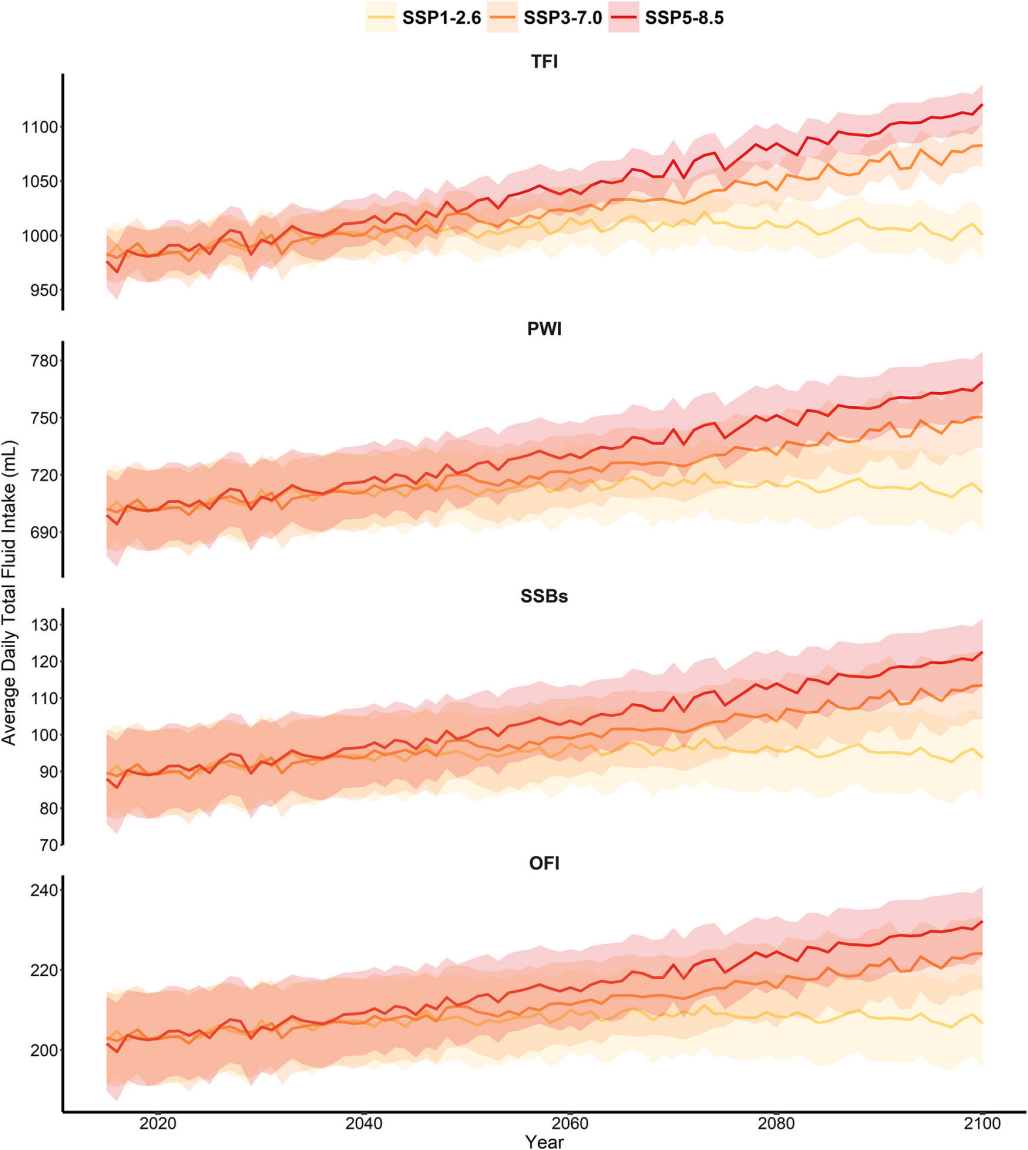
Projected fluid consumption from September to November during 2015–2099 under different SSP scenarios. TFI total fluid intake, PWI plain water intake, SSBs sugar sweetened beverages, OFI other fluid intake, SSP shared socioeconomic pathways. Projections are based on climate scenarios SSP1-2.6, SSP3-7.0, and SSP5-8.5, representing low, medium, and high greenhouse gas emission trajectories, respectively. All underlying data can be found in Supplementary Data 4; Shaded area denotes the 95% confidence interval of the estimated parameter.

Figure 5 presents the estimated fluid consumption in various periods under the SSP1-2.6, SSP3-7.0, and SSP5-8.5 scenarios. Daily fluid consumption is projected to increase from 991 mL (95% CI: 968–1014 mL) in the 2020 s to 1107 mL (95% CI: 9798–1126 mL) by the 2090 s under the SSP585 scenario.

Whereas the gradient gradually increased under SSP126, rising from 988 mL (95% CI: 964–1012 mL) in the 2020 s to 1011 mL (95% CI: 989–1033 mL) in the 2060 s and then decreased to 1005 mL (95% CI: 983–1027 mL) in the 2090 s. Meanwhile, the same trend was observed among males and females (Supplementary Fig. 4), as well as among various age groups (Supplementary Fig. 5).

### Robustness validation through sensitivity analysis

Varying the *df* for RH to 2, 4, and 5, as well as excluding outliers, demonstrated stability in both TFI and PWI (Supplementary Figs. 6-9). Additionally, replacing the *ns* function in the cross-basis framework with a linear model yielded consistent results, further supporting the robustness of the findings (Supplementary Fig. 10).

## Discussion

Based on 24-h fluid survey data collected from primary and middle school students in 2011, our study revealed that ambient temperature was near linearity positively associated with various types of fluid consumption. In addition, we projected a notable increase in fluid consumption during the 2099 under the SSP585 scenario, reflecting the potential impact of rising temperatures on hydration needs. Our findings have important implications for TFI recommendations and contribute to understanding fluid consumption dynamics in the context of global warming.

The median daily TFI consumption of students from the three cities was 1064 mL, indicating a lower likelihood of meeting the recommended fluid intake guidelines^10^. This finding aligned with the most recent national cross-sectional survey on fluid intake in urban China, with daily TFI among children and adolescents of 966 and 1177 mL, respectively^9^. The insufficient fluid intake may be attributed to several factors, including a lack of awareness regarding hydration needs, limited access to water during certain periods of the day. While structured school schedules provide stability and access to water during weekdays, they may simultaneously limit drinking opportunities during class hours. Younger children, in particular, may not adequately respond to thirst signals, further contributing to underhydration in certain settings.

In our study, we found that children and adolescents chose healthier PWI and fewer SSBs. Almost up to 60% of the TFI consumed was PWI, consistent with a systematic review that water contributed up to 58% and 75% of TFI among children and adolescents^34^. The preference for PWI over SSBs in younger children may be partly explained by school environments where PWI is the primary beverage provided and by economic factors, as younger children generally have less access to pocket money needed to purchase SSBs from external sources^35^. However, the consumption of SSBs displayed age-dependent variations, with higher consumption among high school students. Meanwhile, primary school students exhibited higher MDI consumption^9,34^. The availability of beverages, whether in school or at home, plays a critical role in shaping fluid intake behaviors, with weekday and weekend differences further influencing these patterns. Consistent with previous research on primary and middle school students^7,9^, in the present study, TFI exhibited a gradual increase with age and displayed sex differences, which might be linked to increased physical activity levels and possibly greater awareness of hydration needs among older students. The higher fluid consumption among urban students may stem from greater accessibility to water sources and potentially higher awareness of the importance of hydration.

A positive association was observed between ambient temperature and fluid intake, specifically TFI and PWI, consistent with previous studies. Each 1 °C rise in temperature corresponded to an increase of 24 mL in daily TFI and 12 mL in daily PWI, driven by enhanced perspiration, evaporation, and respiratory water loss^11–13,28,29^. While the observed exposure-response relationship appeared near-linear, fluid intake required for health and thermoregulation may rise non-linearly at higher temperatures, potentially exceeding the values observed and projected in our study. Under such conditions, PWI alone may be insufficient to replace ions lost through sweat, necessitating the inclusion of electrolyte-containing beverages in future hydration guidelines, particularly in hotter climates or during prolonged physical activity.

In this study, SSBs consumption increased by 8.45%, marking the highest relative increment among all types of fluid intake. Rising temperatures may stimulate an augmented desire for cold beverages, offering heightened refreshment and flavor. SSBs, being widely accessible, provide sweetened and flavored alternatives that are perceived as gratifying and thirst-quenching due to their cold and convenient packaging^36^. Additionally, warmer temperatures can encourage outdoor activities, where the consumption of SSBs is often prevalent. However, the increased reliance on SSBs poses notable health risks. A substantial body of evidence has established a strong association between consistent consumption of SSBs and adverse health outcomes such as weight gain, a higher risk of type 2 diabetes mellitus, cardiovascular diseases, and certain forms of cancer^37^. To address this trend, it is critical to prioritize healthier hydration strategies, including the incorporation of electrolyte-containing beverages^38^. As elevated temperatures drive higher fluid loss through sweat, these beverages are essential for replacing not only water but also critical electrolytes such as sodium, potassium, and magnesium, which are crucial for maintaining physiological balance and preventing dehydration-related complications.

Stratified analyses revealed that temperature had a greater impact on daily TFI among high school students (16–18 years). Adolescents tend to engage in more outdoor activities and sports, especially in warmer weather, leading to increased fluid loss through sweating^39^. Furthermore, the effect of temperature increases on daily TFI consumption was much greater in Beijing and Shanghai, exhibiting 3.6 and 2.7 times greater impact, respectively, than in Guangzhou. This regional difference may result from different temperatures among the cities, influenced by variations in elevation. The association between temperature and TFI also indicated a steeper increase at lower temperatures than at higher temperatures, making Guangzhou, with its consistently high temperatures less susceptible to temperature changes than Shanghai and Beijing. We also found that temperature had a greater impact on daily fluid intake during weekdays compared to weekends. This may be attributed to the structured routines and consistent water availability in schools, which promote regular hydration. In contrast, weekends are less structured, with varied activities and access to water, leading to irregular fluid intake patterns^40^.

Our projections indicate that fluid consumption will increase due to global warming. Rising temperatures will accelerate evaporation from land surfaces, reducing streamflow volumes and water availability^41^. In addition, climate change-induced reductions in glaciers would ultimately reduce baseflow in the long term. The 11.7% increase in TFI, though modest at an individual level, represents substantial water resource demands at a population scale, especially in water-stressed regions^42,43^. Combined with societal growth, urbanization, and climate change, this trend will exacerbate water scarcity. According to the WHO, a total of 1.9 million (3.3%) global deaths and 123 million (4.6%) global disability-adjusted life years (DALYs) were attributed to inadequate/unsanitary fresh water in 2016. Children under 5 years old were the most vulnerable population (13% deaths and 12% DALYs), and Sub-Saharan Africa was the most sensitive region (53% deaths and 60% DALYs)^44^. The United Nations World Water Development Report 2018 estimated that 3.6 billion of the world’s population lived in areas that suffered potential water scarcity at least one month per year, and this number could grow to 4.8–5.7 billion by 2050^45^. It is worth noting that our projection model assumes no significant changes in behavior in response to rising temperatures. As temperatures increase, people may spend more time indoors, potentially reducing their need for increased fluid intake. As a water-scarce country, it is necessary to prevent the deteriorating trend of fresh water resources driven by global warming, which will reduce adverse health impacts.

Our study has several limitations. Firstly, the cross-sectional design restricts our ability to infer causality, limiting definitive conclusion about the relationship between temperature and fluid consumption. Additionally, self-reported fluid intake is prone to bias, particularly among younger students who may experience more reporting errors compared to older students. Secondly, this study focused solely on fluid intake and did not encompass dietary water intake from foods, potentially underestimating total water consumption. Thirdly, the data were collected from three cities in September and October, may not fully represent the national student population. This limits the study’s temporal scope and excludes seasonal variations in fluid intake, particularly in hot and humid climates, thus not capturing year-round trends. Additionally, the observational temperature range was limited to 32 °C, reflecting the recorded temperatures during the study period. This does not account for potential non-linear fluid intake responses at higher temperatures, while intensified sweating and insensible perspiration may alter fluid consumption patterns. Extreme environmental conditions, such as hot dry or hot humidity scenarios, were not observed, further limiting the generalizability of our findings. To comprehensively understand the impact of temperature on children’s fluid intake, future research should include diverse geographic regions, various seasons, and a broader range of temperature exposures.

Despite these limitations, our study presents several notable strengths. Firstly, it encompasses a substantial sample size of primary and middle school students across different regions of China, enhancing our findings’ generalizability. Secondly, the comprehensive dataset allowed for detailed analyses, including stratification by age, sex, BMI, and region, thus providing a nuanced understanding of fluid intake patterns in response to temperature changes. Moreover, while this study may underestimate total water consumption by excluding dietary water, the projection of future fluid intake under different climate change scenarios offers valuable insights for policymakers and health professionals in planning and adapting to the anticipated impacts of global warming on children’s hydration needs when developing future guidelines and standards.

In conclusion, our results suggest a positive association between ambient temperature and daily fluid intake among Chinese primary and middle school students, highlighting temperature as a critical consideration for future hydration guidelines. While these findings provide valuable insights, their generalizability may be limited to similar climatic, cultural, and educational contexts. Further studies in diverse geographic and demographic settings are warranted to confirm these associations. Moreover, global warming might increase water requirements and deteriorate water resource shortages in the future, which may lead to serious adverse health risks.

## Supplementary information

Supplementary information

Description of Additional Supplementary Files

Supplementary Data 1-4

Reporting Summary

## Data Availability

The dataset generated and analyzed during this study is available from the corresponding authors upon reasonable request. The source data for Fig. 2 is in Supplementary Data 1. The source data for Fig. 3 is in Supplementary Data 2. The source data for Fig. 4 is in Supplementary Data 3. The source data for Fig. 5 is in Supplementary Data 4.

## Code availability

The code is available on GitHub^46^.
