# Supplementary material for: Projecting future fluid intake of Chinese children in a warming world
Source: Commun Med (Lond). 2025 Jun 3;5:211. doi: 10.1038/s43856-025-00929-0 (PMC12134106; doi:10.1038/s43856-025-00929-0)
Supplement: Supplementary file 1 — Supplementary information [file 43856_2025_929_MOESM1_ESM.pdf]

Projecting future fluid intake of Chinese children in a warming world

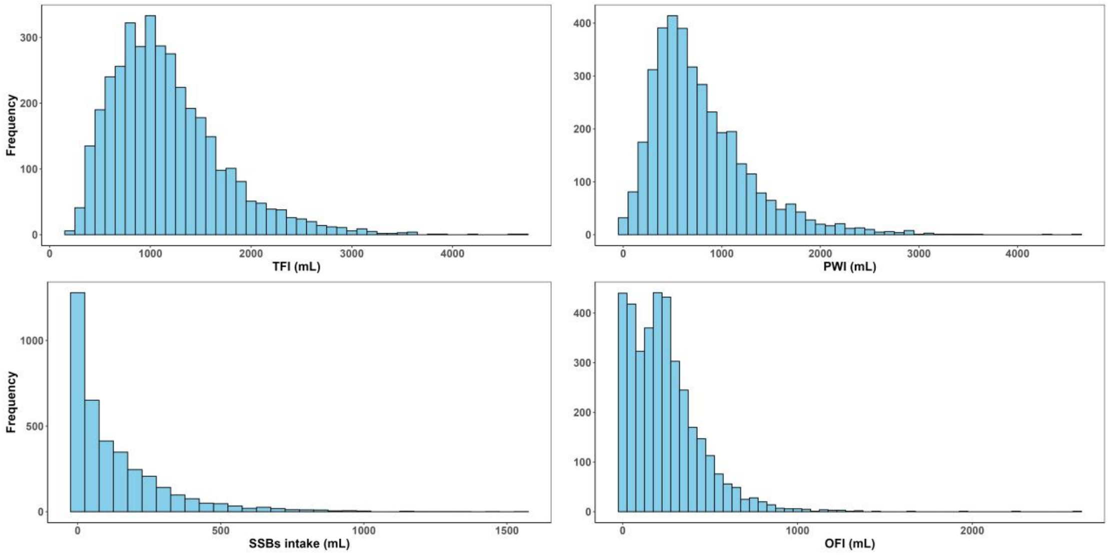

Supplementary Figure 1. The histogram plots of the different types of fluid intake in China. TFI = total fluid intake; PWI = plain water intake; SSBs = Sugar sweetened beverages; OFI = other fluid intake.

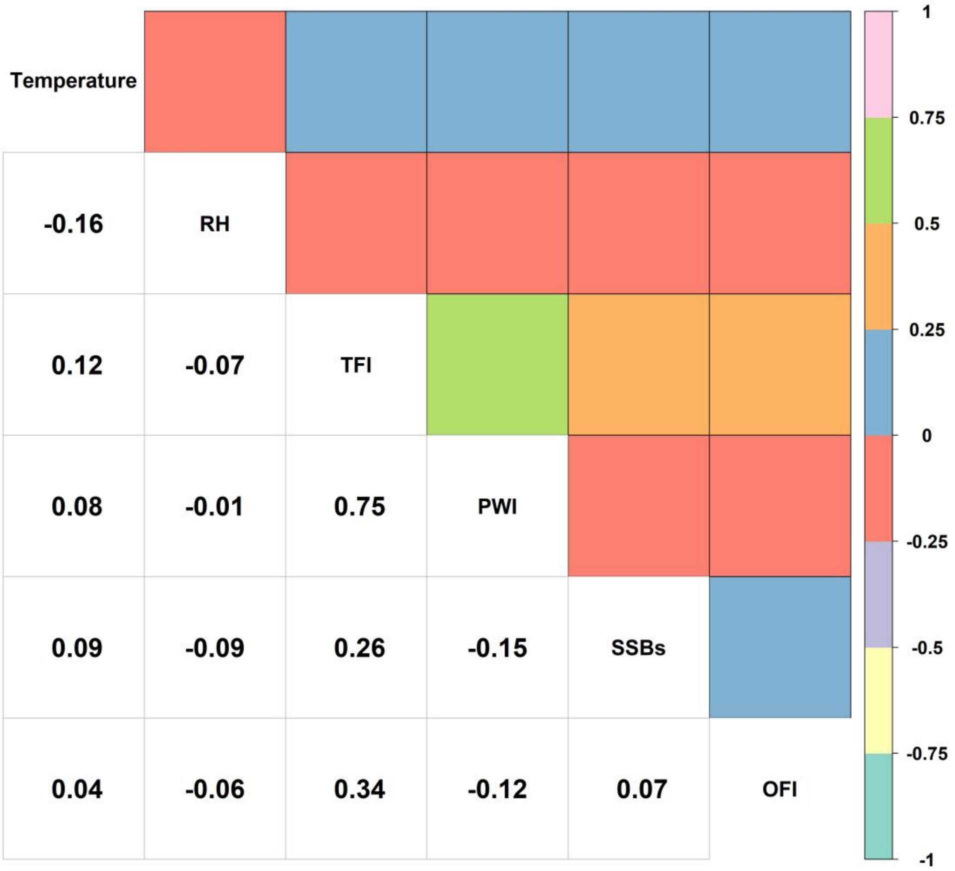

Supplementary Figure 2. The Spearman rank associations between meteorological factors and different types of fluid intakes. Numerical values in the lower-left corner of each cell indicate the Spearman correlation coefficient, quantifying the strength and direction of the association. The color intensity visually represents the magnitude of the correlation, with warmer colors indicating stronger positive correlations and cooler colors indicating stronger negative correlations. RH = relative humidity; TFI = total fluid intake; PWI = plain water intake; SSBs = Sugar sweetened beverages; OFI = other fluid intake.

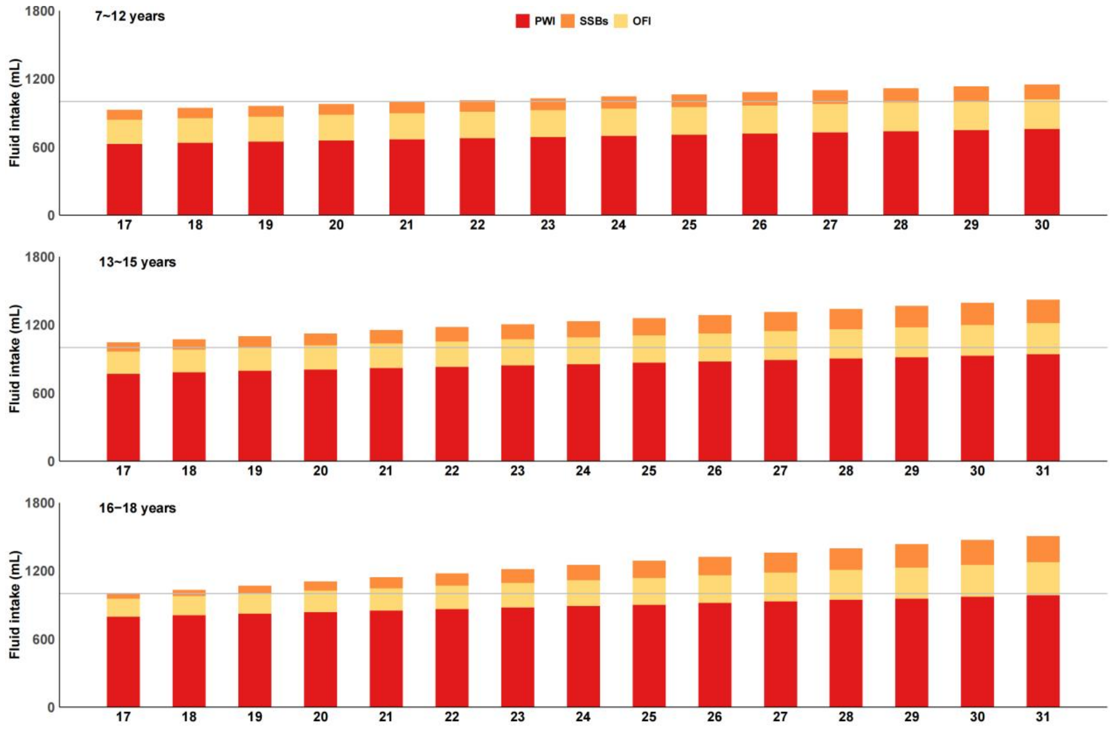

Supplementary Figure 3. Different types of fluid intake at different temperatures, by age groups. Stacked bar charts illustrate the contributions of different fluid types to total daily intake across temperature ranges; The grey horizontal line in the middle represents the 1000 mL reference level for daily fluid intake, providing a visual benchmark for comparison; TFI = total fluid intake; PWI = plain water intake; SSBs = Sugar sweetened beverages; OFI = other fluid intake.

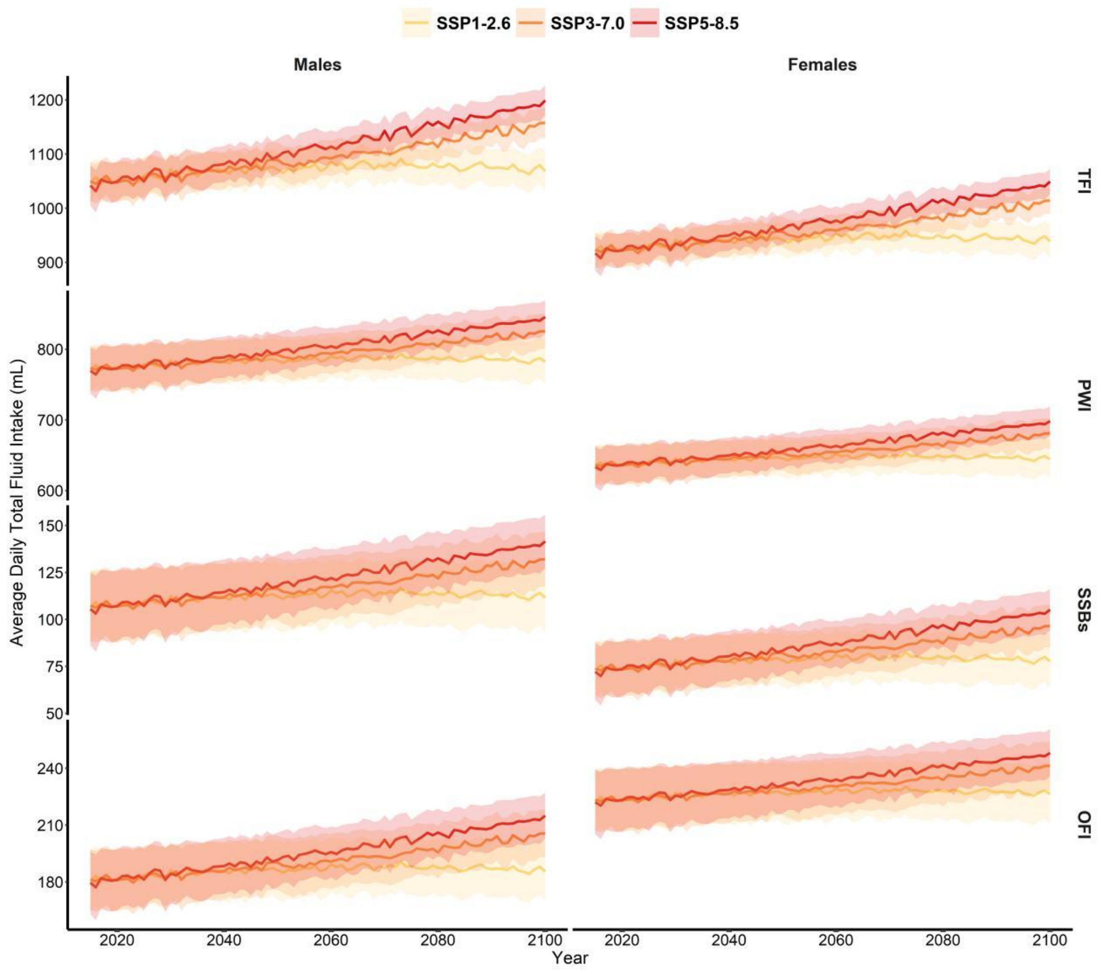

Supplementary Figure 4. Projected fluid consumption from September to November during 2015-2100 under different SSP scenarios, by sex. Projections are based on climate scenarios SSP1-2.6, SSP3-7.0, and SSP5-8.5, representing low, medium, and high greenhouse gas emission trajectories, respectively; Shaded areas represent the 95% confidence intervals of the estimates; SSP = Shared Socioeconomic Pathways; TFI = total fluid intake; PWI = plain water intake; SSBs = Sugar sweetened beverages; OFI = other fluid intake.

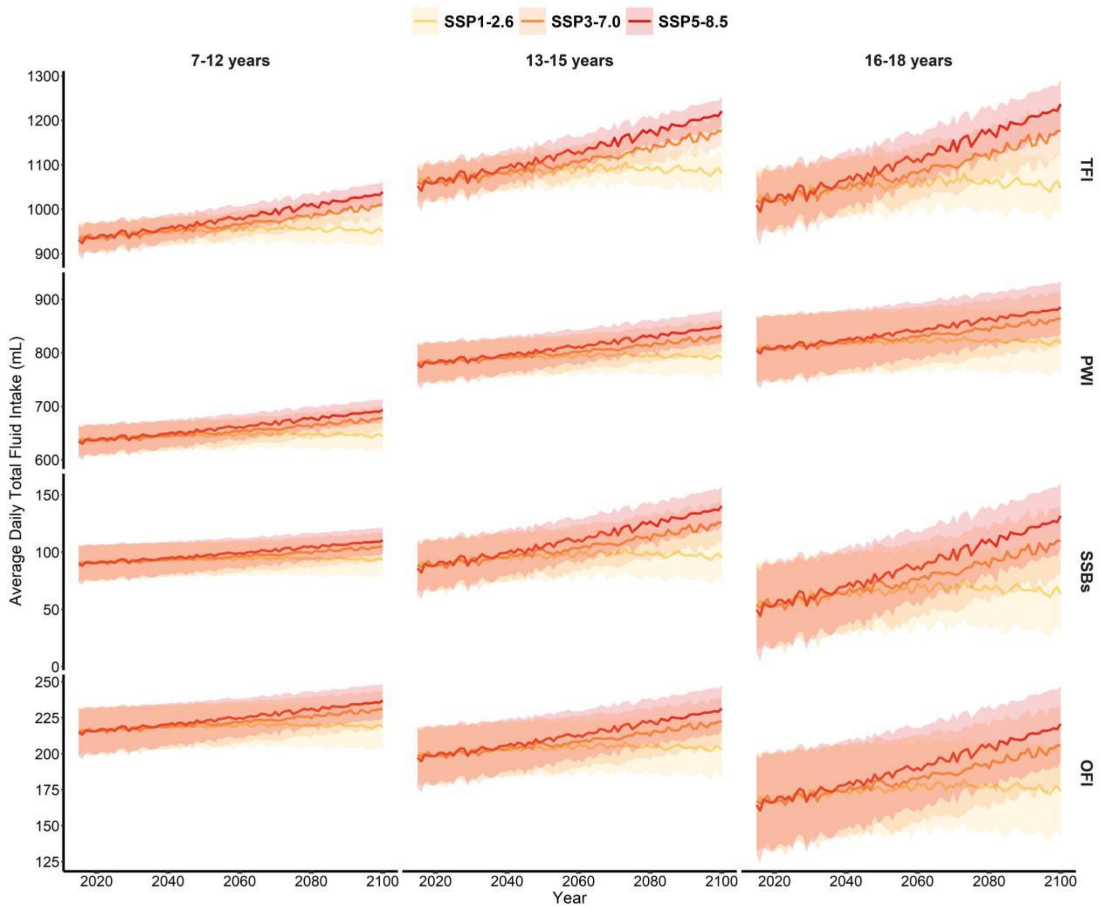

Supplementary Figure 5. Projected fluid consumption from September to November during 2015-2100 under different SSP scenarios by age groups.Projections are based on climate scenarios SSP1-2.6, SSP3-7.0, and SSP5-8.5, representing low, medium, and high greenhouse gas emission trajectories, respectively; Shaded areas represent the 95% confidence intervals of the estimates; SSP = Shared Socioeconomic Pathways; TFI = total fluid intake; PWI = plain water intake; SSBs = Sugar sweetened beverages; OFI = other fluid intake.

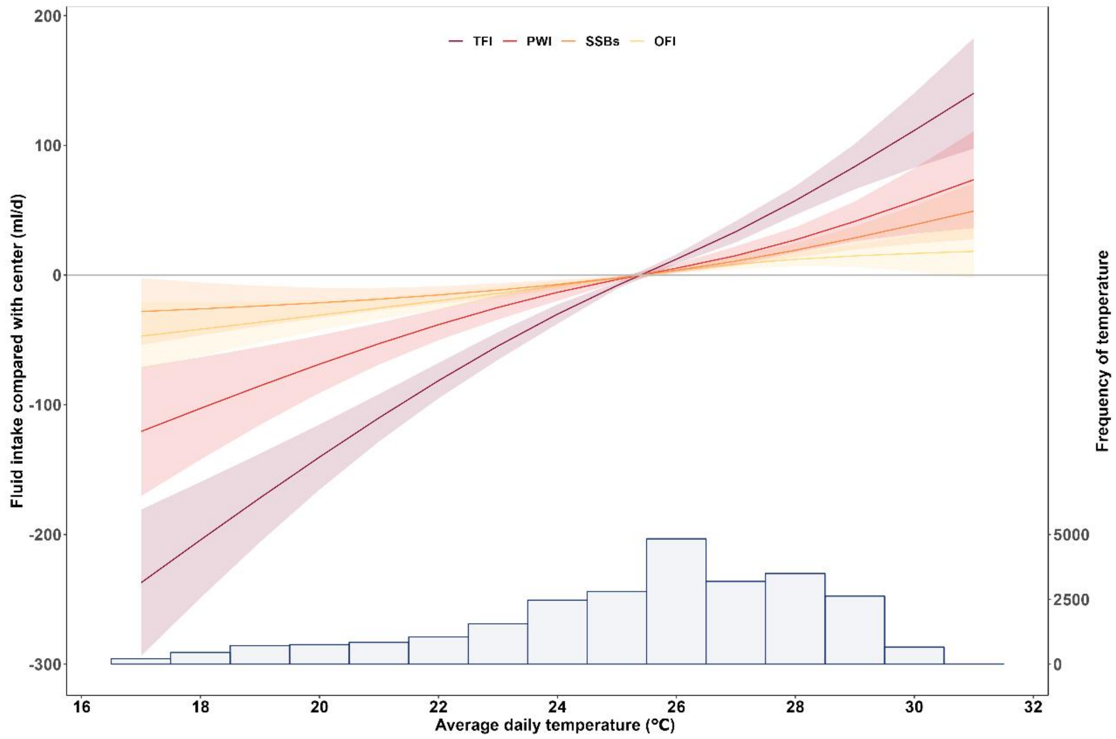

Supplementary Figure 6. The associations between daily mean temperature and 24-h fluid consumption among children across three Chinese cities with adjusted *df* = 2 for RH. Both TFI, PWI and SSBs remained stable, confirming the robustness of the results,while the reduced sample size for OFI caused minor changes. The bar graph at the bottom illustrates the frequency distribution of daily mean temperatures recorded during the study period. df = degree of freedom; RH = relative humidity; TFI = total fluid intake; PWI = plain water intake; SSBs = Sugar sweetened beverages; OFI = other fluid intake; Shaded area denotes the 95% confidence interval of the estimated parameter.

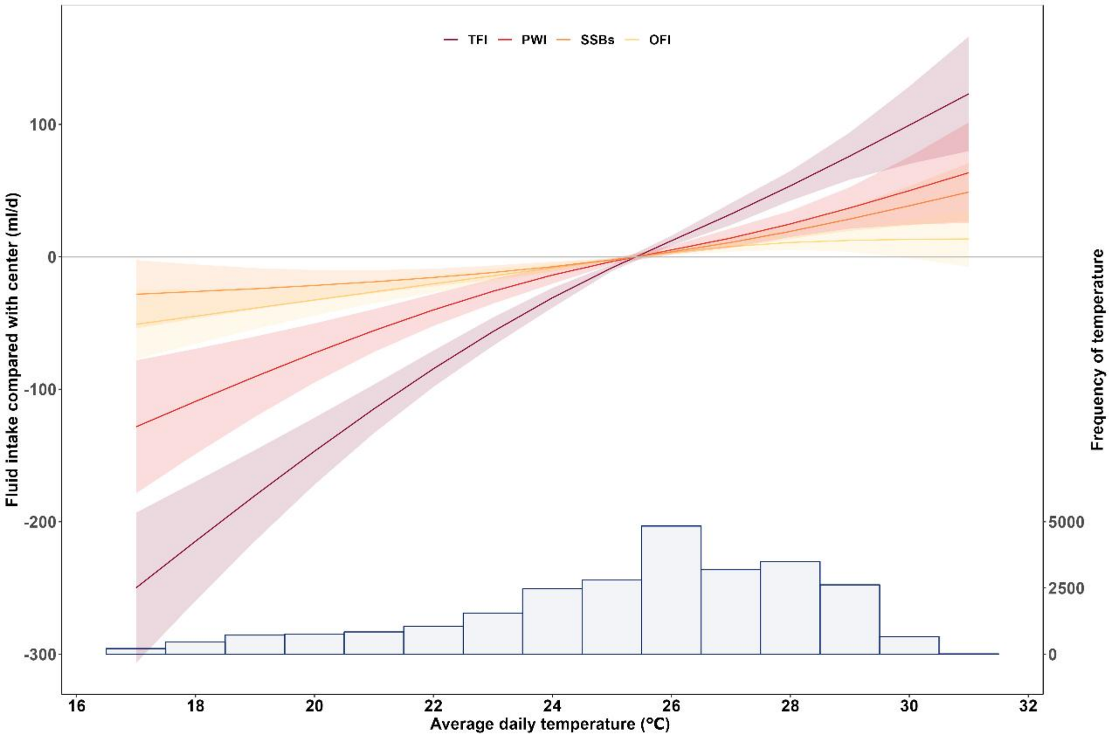

Supplementary Figure 7. The associations between daily mean temperature and 24-h fluid consumption among children across three Chinese cities with adjusted *df* = 4 for RH. Both TFI, PWI and SSBs remained stable, confirming the robustness of the results,while the reduced sample size for OFI caused minor changes. The bar graph at the bottom illustrates the frequency distribution of daily mean temperatures recorded during the study period. df = degree of freedom; RH = relative humidity; TFI = total fluid intake; PWI = plain water intake; SSBs = Sugar sweetened beverages; OFI = other fluid intake; Shaded area denotes the 95% confidence interval of the estimated parameter.

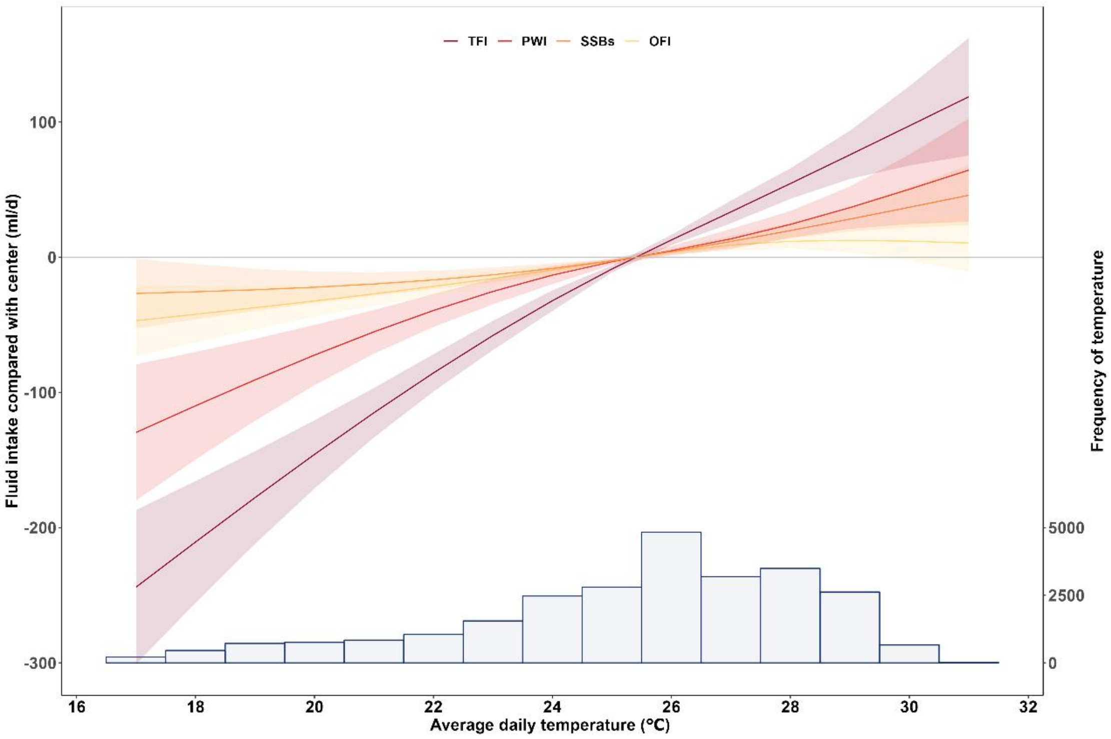

Supplementary Figure 8. The associations between daily mean temperature and 24-h fluid consumption among children across three Chinese cities with adjusted *df* = 5 for RH. Both TFI, PWI and SSBs remained stable, confirming the robustness of the results,while the reduced sample size for OFI caused minor changes. The bar graph at the bottom illustrates the frequency distribution of daily mean temperatures recorded during the study period. df = degree of freedom; RH = relative humidity; TFI = total fluid intake; PWI = plain water intake; SSBs = Sugar sweetened beverages; OFI = other fluid intake; Shaded area denotes the 95% confidence interval of the estimated parameter.

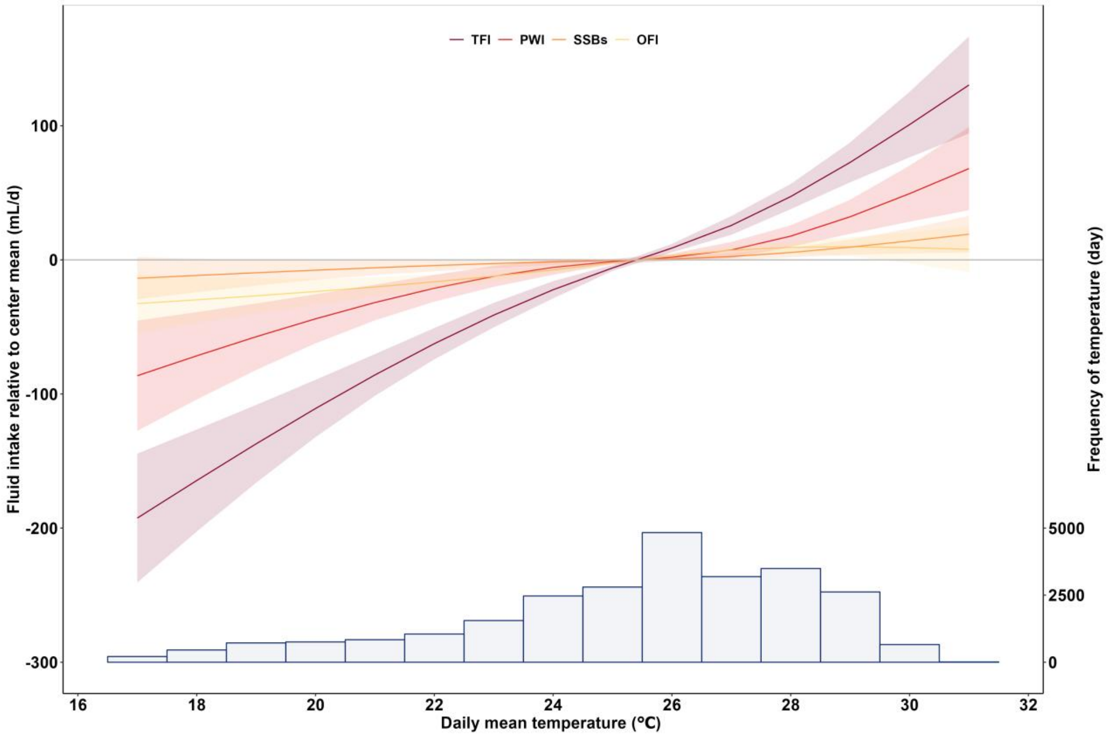

Supplementary Figure 9. The associations between daily mean temperature and 24-h fluid consumption among children across three Chinese cities, excluding outliers. Both TFI and PWI remained stable, confirming the robustness of the results, while the reduced sample size for SSBs and OFI caused minor changes. The bar graph at the bottom illustrates the frequency distribution of daily mean temperatures recorded during the study period. TFI = total fluid intake; PWI = plain water intake; SSBs = Sugar sweetened beverages; OFI = other fluid intake; Shaded area denotes the 95% confidence interval of the estimated parameter.

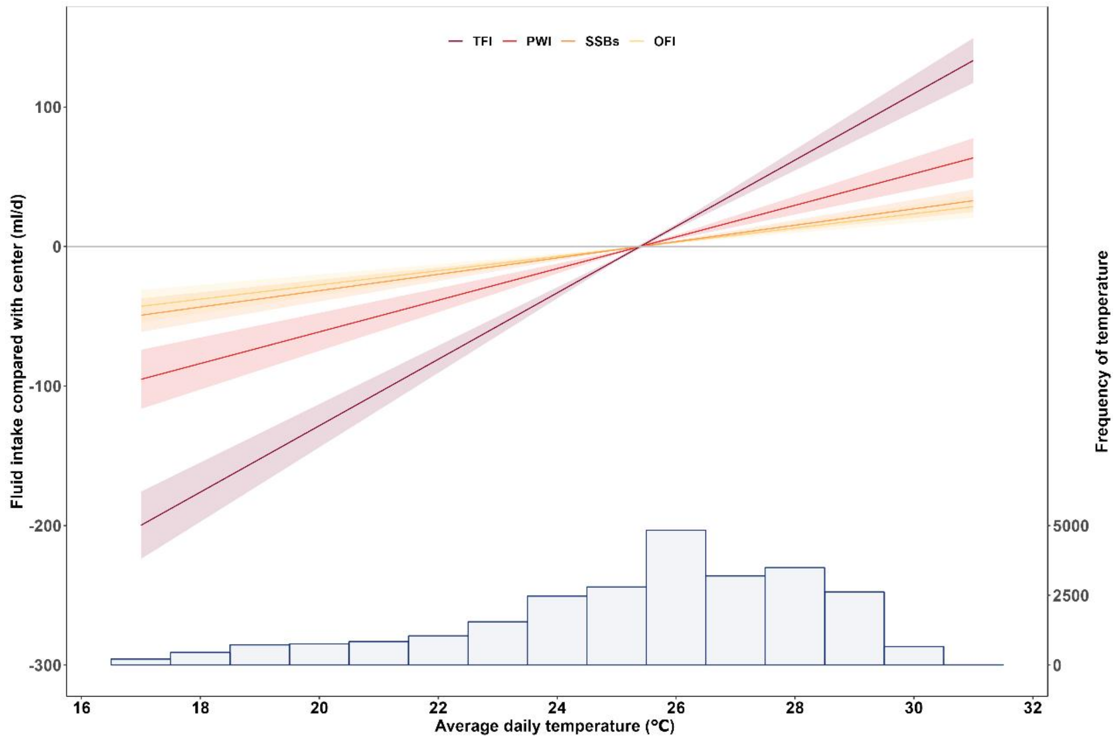

Supplementary Figure 10. The associations between daily mean temperature and 24-h fluid consumption among children across three Chinese cities, incorporating an adjusted linear function within the cross-basis framework for temperature. The results remained consistent. The bar graph at the bottom illustrates the frequency distribution of daily mean temperatures recorded during the study period. TFI = total fluid intake; PWI = plain water intake; SSBs = Sugar sweetened beverages; OFI = other fluid intake; Shaded area denotes the 95% confidence interval of the estimated parameter.

Supplementary Table 1

Median daily consumption of other sources of fluid intake by characteristics and socioeconomic status of students in China (mL).

Variables	CSDS		SSDs		FBI		HBI		CBI		MDI		Others	
	Median	IQR	Median	IQR	Median	IQR	Median	IQR	Median	IQR	Median	IQR	Median	IQR
Total	0	64	0	93	0	0	0	0	0	0	171	212	0	0
Sex														
Male	0	86	0	93	0	0	0	0	0	0	157	228	0	0
Female	0	43	21	94	0	0	0	21	0	0	179	200	0	14
Age group (Years)														
7~12	0	57	0	71	0	0	0	0	0	0	171	205	0	0
13~15	0	76	7	107	0	0	0	0	0	0	168	228	0	0
16~18	0	50	36	138	0	0	0	29	0	0	159	206	0	0
BMI group														
Wasting	0	64	0	93	0	0	0	0	0	0	164	193	0	0
Standard	0	64	0	99	0	0	0	0	0	0	171	213	0	0
Overweight	0	71	0	78	0	0	0	0	0	0	179	217	0	0
Obesity	0	59	0	98	0	0	0	0	0	0	153	216	0	0
Region														
Urban	0	57	17	109	0	0	0	17	0	0	179	216	0	0
Rural	0	72	0	71	0	0	0	0	0	0	150	200	0	0
Physical activity														

Low	0	57	0	86	0	0	0	0	0	0	147	203	0	0
Moderate	0	64	0	88	0	0	0	0	0	0	174	210	0	0
High	0	71	14	101	0	0	0	14	0	0	193	218	0	0
Household income (RMB)														
<2000	0	68	0	57	0	0	0	0	0	0	126	200	0	0
2000~3999	0	71	0	86	0	0	0	0	0	0	143	200	0	0
4000~5999	0	71	0	82	0	0	0	0	0	0	171	206	0	0
6000-7999	0	57	0	96	0	0	0	0	0	0	163	214	0	0
≥8000	0	64	29	107	0	0	0	14	0	0	193	213	0	0
Prepared water														
No	0	57	7	109	0	0	0	0	0	0	178	214	0	0
Yes	0	69	0	89	0	0	0	0	0	0	170	212	0	0
City														
Beijing	0	51	29	121	0	0	0	29	0	0	166	202	0	14
Shanghai	0	64	21	114	0	0	0	14	0	0	211	207	0	0
Guangzhou	0	71	0	71	0	0	0	0	0	0	130	200	0	0
Weekday														
No	0	14	0	29	0	0	0	0	0	0	43	75	0	0
Yes	0	36	0	69	0	0	0	0	0	0	121	157	0	0

Abbreviations: IQR, interquartile range; CSDs, carbonated soft drinks; SSDs, still sugary drinks; FBI, functional beverage intake; HBI, hot beverages intake; CBIs, cold beverage intake; MDI, milk drinks intake; Others, other beverage.
