# Supplementary material for: Projecting future fluid intake of Chinese children in a warming world
Source: Commun Med (Lond). 2025 Jun 3;5:211. doi: 10.1038/s43856-025-00929-0 (PMC12134106; doi:10.1038/s43856-025-00929-0)
Supplement: Supplementary file 2 — Description of Additional Supplementary Files [file 43856_2025_929_MOESM2_ESM.pdf]

**Supplementary Data 1.** This dataset provides the source data used to generate Fig. 2, illustrating the relationship between daily mean temperature and 24-hour fluid consumption relative to the center mean. tm: Daily mean temperature (°C); WA: Estimated mean 24-hour fluid intake at each temperature point (mL); WAL: Lower bound of the 95% CI for fluid intake (mL); WHA: Upper bound of the 95% CI for fluid intake (mL); group: Type of fluid consumed (TFI: Total Fluid Intake, PWI: Plain Water Intake, SSBs: Sugar-Sweetened Beverages, OFI: Other Fluid Intake).

**Supplementary Data 2.** This dataset provides the source data for Fig. 3, showing estimated changes in fluid intake per 1°C increase in temperature across different subgroups. TIF Changes: Change in Total Fluid Intake (mL); TIF Changes Lower/Upper: 95% CI bounds for TFI change (mL); PWI Changes: Change in Plain Water Intake (mL); PWI Changes Lower/Upper: 95% CI bounds for PWI change (mL); SSBs Changes: Change in Sugar-Sweetened Beverages intake (mL); SSBs Changes Lower/Upper: 95% CI bounds for SSBs change (mL); OFI Changes: Change in Other Fluid Intake (mL); OFI Changes Lower/Upper: 95% CI bounds for OFI change (mL).

**Supplementary Data 3.** This dataset provides the source data for Fig. 4, illustrating fluid intake at different temperatures by sex. tm: Daily mean temperature (°C); type: Population group (ALL, Male, Female); group: Fluid type (PWI: Plain Water Intake, SSBs: Sugar-Sweetened Beverages, OFI: Other Fluid Intake); water_mean: Estimated mean fluid intake (mL); water_low: Lower bound of the 95% CI (mL); water_high: Upper bound of the 95% CI (mL).

**Supplementary Data 4.** This dataset provides the source data for Fig. 5, showing projected fluid

intake trends under different climate scenarios. ssp: Shared Socioeconomic Pathway scenario (SSP1-2.6, SSP3-7.0, SSP5-8.5); high: Upper bound of the 95% CI for fluid intake (mL); low: Lower bound of the 95% CI for fluid intake (mL); mean: Estimated mean fluid intake (mL); water: Fluid type (PWI: Plain Water Intake, SSBs: Sugar-Sweetened Beverages, OFI: Other Fluid Intake, TFI: Total Fluid Intake).
