# Supplementary material for: Projecting future fluid intake of Chinese children in a warming world
Source: Commun Med (Lond). 2025 Jun 3;5:211. doi: 10.1038/s43856-025-00929-0 (PMC12134106; doi:10.1038/s43856-025-00929-0)
Supplement: Supplementary file 4 — Reporting Summary [file 43856_2025_929_MOESM4_ESM.pdf]

nature portfolio

Corresponding author(s): Guangshen Ma, Wenjun Ma

Last updated by author(s): Apr 6, 2025

Reporting Summary

Nature Portfolio wishes to improve the reproducibility of the work that we publish. This form provides structure for consistency and transparency in reporting. For further information on Nature Portfolio policies, see our Editorial Policies and the Editorial Policy Checklist.

Statistics

For all statistical analyses, confirm that the following items are present in the figure legend, table legend, main text, or Methods section.

n/a	Confirmed	
☒	☐	The exact sample size (*n*) for each experimental group/condition, given as a discrete number and unit of measurement
☐	☒	A statement on whether measurements were taken from distinct samples or whether the same sample was measured repeatedly
☐	☒	The statistical test(s) used AND whether they are one- or two-sided<br>*Only common tests should be described solely by name; describe more complex techniques in the Methods section.*
☐	☒	A description of all covariates tested
☐	☒	A description of any assumptions or corrections, such as tests of normality and adjustment for multiple comparisons
☐	☒	A full description of the statistical parameters including central tendency (e.g. means) or other basic estimates (e.g. regression coefficient) AND variation (e.g. standard deviation) or associated estimates of uncertainty (e.g. confidence intervals)
☐	☒	For null hypothesis testing, the test statistic (e.g. *F*, *t*, *r*) with confidence intervals, effect sizes, degrees of freedom and *P* value noted<br>*Give P values as exact values whenever suitable.*
☒	☐	For Bayesian analysis, information on the choice of priors and Markov chain Monte Carlo settings
☐	☒	For hierarchical and complex designs, identification of the appropriate level for tests and full reporting of outcomes
☒	☐	Estimates of effect sizes (e.g. Cohen's *d*, Pearson's *r*), indicating how they were calculated

Our web collection on statistics for biologists contains articles on many of the points above.

Software and code

Policy information about availability of computer code

Data collection	The data were collected via a questionnaire-based survey in 2011, targeting students age 7-18 years from randomly selected schools in urban and rural areas of Beijing, Shanghai, and Guangzhou, excluding those with chronic illnesses.
Data analysis	All results were two-sided, and a p-value less than 0.05 was defined as statistically significant. We used R software version 4.2.3 with the 'lmerTest' package to build a distributed generalized linear mixed effects regression model.

For manuscripts utilizing custom algorithms or software that are central to the research but not yet described in published literature, software must be made available to editors and reviewers. We strongly encourage code deposition in a community repository (e.g. GitHub). See the Nature Portfolio guidelines for submitting code & software for further information.

Data

Policy information about availability of data

All manuscripts must include a data availability statement. This statement should provide the following information, where applicable:

- Accession codes, unique identifiers, or web links for publicly available datasets
- A description of any restrictions on data availability
- For clinical datasets or third party data, please ensure that the statement adheres to our policy

Provide your data availability statement here.

Research involving human participants, their data, or biological material

Policy information about studies with human participants or human data. See also policy information about sex, gender (identity/presentation), and sexual orientation and race, ethnicity and racism.

Reporting on sex and gender	We specifically utilized 'sex' to distinguish biological differences and conducted sex-stratified analyses to explore potential variations in the outcomes.
Reporting on race, ethnicity, or other socially relevant groupings	We use urban and rural classifications as socially constructed categories. These classifications are based on official administrative divisions in China.'Urban' refers to areas designated as urban districts under the national administrative system. 'Rural' refers to towns or villages outside these urban administrative boundaries.
Population characteristics	age group, sex, BMI group?grade, physical activity, monthly household income, city as well as region.
Recruitment	A multistage, stratified, cluster-randomized sampling design was conducted to select subjects. First, Beijing, Shanghai, and Guangzhou cities were selected, and then both an urban and a rural areas within each city were chosen by simple random sampling. In the third stage, two primary schools, two middle schools, and two high schools were randomly selected from the designated areas. During the fourth stage, one class from each grade (spanning from grades 3 to 6 for primary schools, grades 7 to 9 for middle schools, and grades 10 to 11 for high schools) was selected through a randomized process. The date of birth, monthly house hold income, and physical activity were reported by themselves, which may introduce potential bias. However, comprehensive quality control and data validation procedures were implemented to minimized such bias.
Ethics oversight	The Ethical review committee of the national institute for nutrition and food safety, Chinese center for Disease Control and Prevention approved the study protocol.

Note that full information on the approval of the study protocol must also be provided in the manuscript.

Field-specific reporting

Please select the one below that is the best fit for your research. If you are not sure, read the appropriate sections before making your selection.

☐ Life sciences ☐ Behavioural & social sciences ☒ Ecological, evolutionary & environmental sciences

For a reference copy of the document with all sections, see nature.com/documents/nr-reporting-summary-flat.pdf

Ecological, evolutionary & environmental sciences study design

All studies must disclose on these points even when the disclosure is negative.

Study description	The data were collected in a cross-sectional survey of students aged 7-18 years from urban and rural areas in Beijing, Shanghai, and Guangzhou during September-October 2011, using a multstage, stratified, cluster-randomized sampling design, schools and classes were randomly selected, and healthy students participated. Both temperature and fluid intake were measured repeatedly.
Research sample	A total sampe size estimated were aprroximately 4320. 40 students /class*9 grades*2 schools*2 urban-rural areas*3 cities. A total of 3713 students (1783 males and 1930 females) were included in this manuscipt.
Sampling strategy	A multistage, stratified sampling mehtod selected urban and rural areas in Beijing, Shanghai, and Guangzhou. Schools and classes were randomly chosen, including healthy students aged 7-18 (about 40 students per class).
Data collection	Students recorded their 24-hour fluid intake after training, while medical practitioners measured indoor and outdoor temperature and relative humidity at 10:00 AM and 3:00 PM using standardized equipment. Except for household economic status, which was completed by parents, all other questionnaires were self-reported by the children.
Timing and spatial scale	During September and October 2011 in Beijing, Shanghai, and Guangzhou.
Data exclusions	Students with chronic diseases such as diabetes, hypertension, kidney disorders,or liver disease were excluded.
Reproducibility	Our manuscript was a cross-sectional survey, the concept of reproducibility does not apply.
Randomization	Our manuscript was a cross-sectional survey, the randomization does not apply.
Blinding	Our manuscript was a cross-sectional survey, the blinding dose not apply.

Did the study involve field work? ☒ Yes ☐ No

Field work, collection and transport

Field conditions	The field work was conducted during September and October in 2011, with average temperatures of 26.6 degree in Guangzhou, 25.4 degree in Shanghai, and 22.9 degree in Beijing.
Location	Beijing is located at a latitude of 39.9042°N and a longitude of 116.4074°E, with an elevation of approximately 43 meters above the sea level. Shanghai is situated at 31.2304°N, 121.4737□°E, and has an elevation of about 4 m.Guangzhou lies at 23.1291° N□113.2644°E□ with an elevation of around 21 m.□□□
Access & import/export	NA
Disturbance	The study caused minimal disruption, with surveys conducted during breaks and data collection completed under 10 minutes. School staff coordinated schedules to avoid interfering with teaching hours.

Reporting for specific materials, systems and methods

We require information from authors about some types of materials, experimental systems and methods used in many studies. Here, indicate whether each material, system or method listed is relevant to your study. If you are not sure if a list item applies to your research, read the appropriate section before selecting a response.

Materials & experimental systems

n/a	Involved in the study
☒	☐ Antibodies
☒	☐ Eukaryotic cell lines
☒	☐ Palaeontology and archaeology
☒	☐ Animals and other organisms
☒	☐ Clinical data
☒	☐ Dual use research of concern
☒	☐ Plants

Methods

n/a	Involved in the study
☒	☐ ChIP-seq
☒	☐ Flow cytometry
☒	☐ MRI-based neuroimaging

Plants

Seed stocks	NA
Novel plant genotypes	NA
Authentication	NA
